# Supplementary material for: Epidemiology of secondary infection after snakebites in center-west Brazil
Source: PLoS Negl Trop Dis. 2023 Mar 6;17(3):e0011167. doi: 10.1371/journal.pntd.0011167 (PMC10019779; doi:10.1371/journal.pntd.0011167)
Supplement: S1 Table — (DOCX) [file pntd.0011167.s003.docx]

**Supporting information**

**Supplementary Table 1.** Sociodemographic characteristics of 326 patients treated for snakebites at the State Hospital for Tropical Diseases, Goiás, Brazil, from 2018 to 2019.

|  | **N** | **%** |
| --- | --- | --- |
| **Median age, years (interquartile range)** | 38 (23–54) |  |
| <18 years | 56 | 17.18 |
| 18–60 years | 218 | 66.87 |
| ≥60 years | 52 | 15.95 |
| **Sex** |  |  |
| Men | 252 | 77.3 |
| Women | 74 | 22.7 |
| **Race** |  |  |
| Brown | 282 | 86.5 |
| White | 23 | 7.1 |
| Black | 10 | 3.1 |
| Asian | 8 | 2.4 |
| Ignored | 3 | 0.9 |
| **Education** |  |  |
| Illiterate | 10 | 3.1 |
| 1st–4th grade of Elementary School | 56 | 17.2 |
| 5th–8th grade of Elementary School | 64 | 19.6 |
| High school | 61 | 18.7 |
| College education | 11 | 3.4 |
| Ignored | 110 | 33.7 |
| Not applicable | 14 | 4.3 |
| **Zone of occurrence** |  |  |
| Rural | 215 | 65.9 |
| Urban | 77 | 23.6 |
| Peri-urban | 6 | 1.8 |
| Ignored | 28 | 8.6 |
| **Work related** | 45 | 13.8 |
| **Snakebite type** |  |  |
| Bothropic | 268 | 82.2 |
| Crotalic | 56 | 17.2 |
| Elapid | 1 | 0.31 |
| Indeterminate | 1 | 0.31 |
| **Bite site** |  |  |
| Head | 1 | 0.31 |
| Arm | 8 | 2.46 |
| Hand | 66 | 20.24 |
| Leg | 100 | 30.67 |
| Foot | 149 | 45.7 |
| Ignored | 2 | 0.61 |
| **Time between snakebite and medical care** |  |  |
| 0–1 h | 30 | 9.2 |
| 1–3 h | 78 | 23.9 |
| 3–6 h | 83 | 25.5 |
| 6–12 h | 58 | 17.8 |
| 12–24 h | 25 | 7.7 |
| >24 h | 51 | 15.6 |
| Ignored | 1 | 0.3 |
| **Severity classification** |  |  |
| Mild | 107 | 32.8 |
| Moderate | 129 | 39.8 |
| Severe | 90 | 27.6 |
