# Supplementary material for: Epidemiology of secondary infection after snakebites in center-west Brazil
Source: PLoS Negl Trop Dis. 2023 Mar 6;17(3):e0011167. doi: 10.1371/journal.pntd.0011167 (PMC10019779; doi:10.1371/journal.pntd.0011167)
Supplement: S2 Table — (DOCX) [file pntd.0011167.s004.docx]

**Supporting information**

**Supplementary Table 2.** Characterization of clinical presentations on admission and associated complications according to snakebite type.

| ­ | **Bothropic**  **(n = 268)** | | **Crotalic**  **(n = 56)** | | **Elapid**  **(n = 1)** | | **Total**  **(n = 325*)** | |
| --- | --- | --- | --- | --- | --- | --- | --- | --- |
|  | **N** | **%** | **n** | **%** | **N** | **%** | **N** | **%** |
| **Local presentations** |  |  |  |  |  |  |  |  |
| Pain | 264 | 98.5 | 50 | 89.3 | 1 | 100 | 315 | 96.9 |
| Edema | 249 | 92.9 | 24 | 42.9 | 1 | 100 | 274 | 84.3 |
| Ecchymosis | 53 | 19.8 | 6 | 10.7 | 0 | 0 | 59 | 18.2 |
| Erythema | 51 | 19 | 4 | 7.1 | 0 | 0 | 55 | 16.9 |
| Paresthesia | 26 | 9.7 | 20 | 35.7 | 0 | 0 | 46 | 14.2 |
| Blister | 29 | 10.8 | 0 | 0 | 0 | 0 | 29 | 8.9 |
| Bleeding | 24 | 9 | 2 | 3.6 | 0 | 0 | 26 | 8 |
| Necrosis | 14 | 5.2 | 0 | 0 | 0 | 0 | 14 | 4.3 |
| Heat | 11 | 4.1 | 0 | 0 | 0 | 0 | 11 | 3.4 |
| Pruritus | 3 | 1.1 | 0 | 0 | 0 | 0 | 3 | 0.9 |
| Cyanosis | 3 | 1.1 | 0 | 0 | 0 | 0 | 3 | 0.9 |
| **Systemic signs and symptoms** |  |  |  |  |  |  |  |  |
| Vagal | 72 | 26.9 | 17 | 30.4 | 1 | 100 | 90 | 27.7 |
| Neuroparalytic | 36 | 13.4 | 45 | 80.4 | 1 | 100 | 82 | 25.2 |
| Myolytic | 25 | 9.3 | 33 | 58.9 | 1 | 100 | 59 | 18.2 |
| Bleeding | 16 | 6 | 2 | 3.6 | 0 | 0 | 18 | 5.5 |
| Fever | 13 | 4.9 | 1 | 1.8 | 0 | 0 | 14 | 4.3 |
| Headache | 10 | 3.7 | 3 | 5.4 | 0 | 0 | 13 | 4 |
| Vertigo | 3 | 1.1 | 5 | 8.9 | 0 | 0 | 8 | 2.5 |
| Dyspnea | 2 | 0.7 | 4 | 7.1 | 1 | 100 | 7 | 2.2 |
| **Systemic complications** |  |  |  |  |  |  |  |  |
| Acute kidney injury | 44 | 16.4 | 20 | 35.7 | 0 | 0 | 64 | 19.7 |
| Sepsis | 5 | 1.9 | 1 | 1.8 | 0 | 0 | 6 | 1.8 |
| ARF/APE | 3 | 1.1 | 2 | 3.6 | 0 | 0 | 5 | 1.5 |
| Mechanical ventilation | 2 | 0.7 | 2 | 3.6 | 0 | 0 | 4 | 1.2 |
| Shock | 3 | 1.1 | 1 | 1.8 | 0 | 0 | 4 | 1.2 |
| Myocarditis | 0 | 0.0 | 2 | 3.6 | 0 | 0 | 2 | 0.6 |
| DIC | 1 | 0.4 | 1 | 1.8 | 0 | 0 | 2 | 0.6 |
| PTE | 2 | 0.7 | 0 | 0.0 | 0 | 0 | 2 | 0.6 |
| CHF | 0 | 0.0 | 1 | 1.8 | 0 | 0 | 1 | 0.3 |
| Pericardial effusion | 1 | 0.4 | 0 | 0.0 | 0 | 0 | 1 | 0.3 |
| Inflammatory acute abdomen | 1 | 0.4 | 0 | 0.0 | 0 | 0 | 1 | 0.3 |
| **Local complications** |  |  |  |  |  |  |  |  |
| **Secondary infection** | **148** | **55.2** | **6** | **10.7** | **1** | **100** | **155** | **47.6** |
| Compartment syndrome | 57 | 21.3 | 2 | 3.6 | 0 | 0 | 59 | 18.2 |
| Extensive necrosis | 8 | 3.0 | 0 | 0.0 | 0 | 0 | 8 | 2.5 |
| Monoplegia in the affected limb | 0 | 0.0 | 1 | 1.8 | 1 | 100 | 2 | 0.6 |
| Osteomyelitis | 2 | 0.7 | 0 | 0.0 | 0 | 0 | 2 | 0.6 |
| Amputation of the affected limb | 1 | 0.4 | 0 | 0.0 | 0 | 0 | 1 | 0.3 |

*One patient with the snake type classified as indeterminate was excluded from these characterizations.

**ARF/APE:** acute respiratory failure; **APE:** acute pulmonary edema; **CHF:** congestive heart failure; **DIC:** disseminated intravascular coagulation; **PTE:** pulmonary thromboembolism.

Vasovagal signs: vomit, diarrhea

Neuroparalytic signs: eyelid ptosis, blurred vision

Myolitic signs: myalgia, anemia, dark urine
