# Supplementary material for: Epidemiology of secondary infection after snakebites in center-west Brazil
Source: PLoS Negl Trop Dis. 2023 Mar 6;17(3):e0011167. doi: 10.1371/journal.pntd.0011167 (PMC10019779; doi:10.1371/journal.pntd.0011167)
Supplement: Attachment — Submitted filename: Cover Letter revised.docx [file pntd.0011167.s005.docx]

To PLOS Neglected Tropical Diseases

Editorial Board

We are pleased to submit a revised version of the manuscript ‘Epidemiology of secondary infection after snakebites in center-west Brazil’. Many thanks indeed for your comments. The suggestions have been very helpful in preparing our revision, which we think is much improved over the original version. A detailed response to specific comments is given in bold interleaved between reviewer comments below.

In addition, we have made several other minor alterations to improve the quality and flow of the text.

Yours sincerely,

João Victor Soares Coriolano Coutinho, Taiguara Fraga Guimarães, Bruno Borges Valente and Lísia Gomes Martins de Moura Tomich.

1.	Method section

(A)	Reviewer #2

- “I suggest that the authors consult the STROBE instrument as a checklist and complete the necessary information for the study, especially the information on the methods as they need more details such as the identified variables, sample calculation, form of poisoning diagnosis, snake identification, accident classification, established treatment, definition of secondary infection adopted, among others.”

**We apologize for not having made it clear enough in the first draft. We have included better detail of the selection of cases that were assessed using the STROBE checklist.**

2.	Results section

(A)	Reviewer #2

- “I suggest a flow of case evaluation to understand the path of 326 total patients, exclusion criteria to reach 155 patients in the study.”

**In attention to the great suggestion, we have added a flowchart to show the selection of patients.**

- “the first table should be a clinical characterization of the 155 patients in the study to understand the context.”

**We apologize for this, and we have and included a more adequate characterization of the 155 patients in a Table 1 and characterization of 326 total pacients in Supplementary Table 1 and 2.**

- “Results should have risk factor analysis or comparative infection and non-infection groups to improve outcomes.”

**We agree and have included this throughout the text and incorporated it in Table 1.**

**We apologize for this, and we have redone the tables.**

3. Discussion section

The retrospective study on the epidemiology of secondary infection after snakebites in west-central Brazil is well written and well conducted and contributes significantly to assess the incidence of secondary infections and to characterize the microbiological profile and empirical therapy. Of the 155 cases that progressed to secondary infections, 48.4% (75) were treated empirically 32 with amoxicillin clavulanate and 41.9% (65) were treated with SMT +TMP, with 32 of 33 these 144 cases requiring a change to a second regimen, and ten of these 32 requiring a third regimen.
Your results are not significantly different from other studies published in the literature.

In the literature, wound infection after a snakebite occurs in 9 to 77 percent of patients.
We recently published several articles on secondary infections and found that the primary bacteria responsible for wound infection were A. hydrophila, M. morganii, S. aureus and P. rettgeri. In our series on secondary infections related to Bothrops lanceolatus (Martinique) and Bothros atrox (French Guiana), the isolated bacteria were Aeromonas hydrophila,  Morganella morganii,  group A Streptococcus, and group B Streptococcus.  Patients were treated empirically with third-generation cephalosporin (or amoxicillin-clavulanate), aminoglycoside, and metronidazole combinations. Outcome was favorable in all patients. we suggested at this time that empiric antibiotic therapy including third-generation cephalosporin should be administered to those patients on hospital admission.

(A) Reviewer #1

- “What other germs have you encountered in the oral cavity of snakes?”

**We are sorry about this, but we did not assess the oral cavity of snakes in this study.**

- “Line 147-148. Why do you think SMT+TMP is one of the treatment options for *A. hydrophila*?”

**Line 168-173:** **TMP-SMX becomes an attractive option for the treatment of secondary infections related to snakebites in developing countries due to its low cost, the possibility of being done orally, and also because it has activity against *Morganella Morganii* (the main pathogen described in secondary infections of snakebites) and Gram-positive bacteria[17,18]. However, to be used empirically for *A. hydrophila*, more regional studies evaluating its sensitivity need to be conducted, and it may be considered in cases of mild infections[17,18].**

- “Do you think that TMS+TMP could be an attractive therapeutic strategy to treat envenomed patients in developing countries affected by *A. hydrophila*?”

**Line 168-173: Line 168-173: TMP-SMX becomes an attractive option for the treatment of secondary infections related to snakebites in developing countries due to its low cost, the possibility of being done orally, and also because it has activity against *Morganella Morganii* (the main pathogen described in secondary infections of snakebites) and Gram-positive bacteria[17,18]. However, to be used empirically for *A. hydrophila*, more regional studies evaluating its sensitivity need to be conducted, and it may be considered in cases of mild infections[17,18].**

4. Conclusions section

(A) Reviewer #2

- “after the result there must be the discussion topic and then the conclusion.”

**We apologize about that, and we have reordered that in the text.**
